# X-ray crystallographic studies of the extracellular domain of the first plant ATP receptor, DORN1, and the orthologous protein from *Camelina sativa*

**DOI:** 10.1107/S2053230X16014278

**Published:** 2016-09-22

**Authors:** Zhijie Li, Sayan Chakraborty, Guozhou Xu

**Affiliations:** a Department of Molecular and Structural Biochemistry, North Carolina State University, 26 Polk Hall, Raleigh, NC 27695, USA

**Keywords:** plant ATP receptor, DORN1, lectin receptor kinase I.9, glycosylation, *Arabidopsis thaliana*, *Camelina sativa*

## Abstract

Does not respond to nucleotides 1 (DORN1) has recently been identified as the first membrane-integral plant ATP receptor, which is required for ATP-induced calcium response, mitogen-activated protein kinase activation and defense responses in *Arabidopsis thaliana*. In order to understand DORN1-mediated ATP sensing and signal transduction, crystallization and preliminary X-ray studies were conducted on the extracellular domain of DORN1 (atDORN1-ECD) and that of an orthologous protein, *Camelina sativa* lectin receptor kinase I.9 (csLecRK-I.9-ECD or csI.9-ECD). A variety of deglycosylation strategies were employed to optimize the glycosylated recombinant atDORN1-ECD for crystallization. In addition, the glycosylated csI.9-ECD protein was crystallized at 291 K. X-ray diffraction data were collected at 4.6 Å resolution from a single crystal. The crystal belonged to space group *C*222 or *C*222_1_, with unit-cell parameters *a* = 94.7, *b* = 191.5, *c* = 302.8 Å. These preliminary studies have laid the foundation for structural determination of the DORN1 and I.9 receptor proteins, which will lead to a better understanding of the perception and function of extracellular ATP in plants.

## Abstract 2

The extracellular domain of the *A. thaliana* ATP receptor DORN1 has been expressed and purified; a protein orthologous to DORN1 from *C. sativa* has been crystallized and its X-ray diffraction data have been collected.

## 1. Introduction   

Adenosine 5′-triphosphate (ATP), which is the most important mediator in intracellular energy metabolism (Knowles, 1980 ▸; Engel’Gardt & Lisovskaia, 1955 ▸; Harrison & Maitra, 1968 ▸), is also known to function as an essential extracellular signaling molecule in cell-to-cell communication (Burnstock, 1972 ▸; Webb *et al.*, 1993 ▸). In animals, ATP can either be released from cells into the extracellular matrix by multiple channels or transporters or by exocytosis (Schwiebert & Zsembery, 2003 ▸; Bodin & Burnstock, 2001 ▸; Dutta *et al.*, 2002 ▸; Lazarowski *et al.*, 2003 ▸). It can also be produced by the F_o_F_1_-ATP synthase complex (Mangiullo *et al.*, 2008 ▸). The known animal membrane-associated ATP receptors are either P2X (Kaczmarek-Hájek *et al.*, 2012 ▸) or P2Y receptors (Abbracchio *et al.*, 2006 ▸). P2X receptors are a family of trimeric ligand-gated ion channels (Kawate *et al.*, 2009 ▸), while P2Y receptors are G protein-coupled receptors (GPCRs; Zhang *et al.*, 2014 ▸; Erb *et al.*, 2006 ▸). Both receptors are activated upon ATP binding in several physiological processes, such as fast excitatory neurotransmission, developmental processing, pulmonary function, nociception, auditory and ocular function, the apoptotic cascade, astroglial cell function, metastasis formation, bone and cartilage disease, and platelet aggregation/hemostasis (Khakh & North, 2006 ▸; Polosa & Holgate, 2006 ▸; Jacobson & Gao, 2006 ▸; Khakh & Burnstock, 2009 ▸).

The role of extracellular ATP (eATP) in plant signaling was first proposed in the closure of the Venus flytrap (Jaffe, 1973 ▸). Since then, eATP has been implicated in a variety of plant processes, such as root-hair growth, stress responses, gravitropism, cell viability, pathogen responses and thigmotropism (Tanaka *et al.*, 2010 ▸). These hypotheses have recently been strongly corroborated by the identification of the first plant membrane-integral ATP receptor, DORN1/LecRK-I.9 (Choi *et al.*, 2014 ▸), in *Arabidopsis*. In contrast to the known animal ATP receptors, which are either membrane-embedded protein channels or GPCRs, the DORN1 receptor is a typical membrane-integral receptor kinase. In DORN1, the extracellular ATP-binding domain is a legume-type lectin protein, which belongs to a large family of homologous carbohydrate-binding proteins that are mainly present in the seeds of most legume plants (Barondes, 1988 ▸). The carbohydrate-binding activity of legume lectins entails the presence of metal ions, the binding of which is mediated by a number of metal-binding residues that are conserved in all legume lectin structures (Hervé *et al.*, 1999 ▸; Loris *et al.*, 1998 ▸). However, these residues are missing in the extracellular domain of DORN1. In addition to carbohydrate binding, some legume lectins also recognize adenine or adenine-related plant hormones (Shetty *et al.*, 2013 ▸; Hamelryck *et al.*, 1999 ▸). Adenine is part of ATP; however, it does not compete with ATP for binding to DORN1 (Choi *et al.*, 2014 ▸). Upon ATP binding, the intra­cellular DORN1 kinase domain is presumably activated, resulting in several cellular responses such as mitogen-activated kinase activation, increased cytosolic calcium concentration and induction of gene expression (Cao *et al.*, 2014 ▸). How ATP perception in DORN1 leads to activation of its kinase activity as yet remains unknown.

We initiated the elucidation of the mechanism of DORN1-mediated ATP perception and signaling by determining the crystal structure of the extracellular domain of the *A. thaliana* DORN1 protein (atDORN1-ECD). In addition, we have also conducted a preliminary X-ray analysis of an orthologous protein to DORN1: I.9 from *Camelina sativa* (csI.9-ECD; NCBI Reference Sequence XP_010443820.1). Our undertaking has laid the foundation for structural determination of the atDORN1 and csI.9 receptor proteins, which will lead to a better understanding of the perception and function of eATP in plants.

## 2. Materials and methods   

### 2.1. Macromolecule production   

#### 2.1.1. Protein expression and purification of atDORN1-ECD   

**Figure 1:**
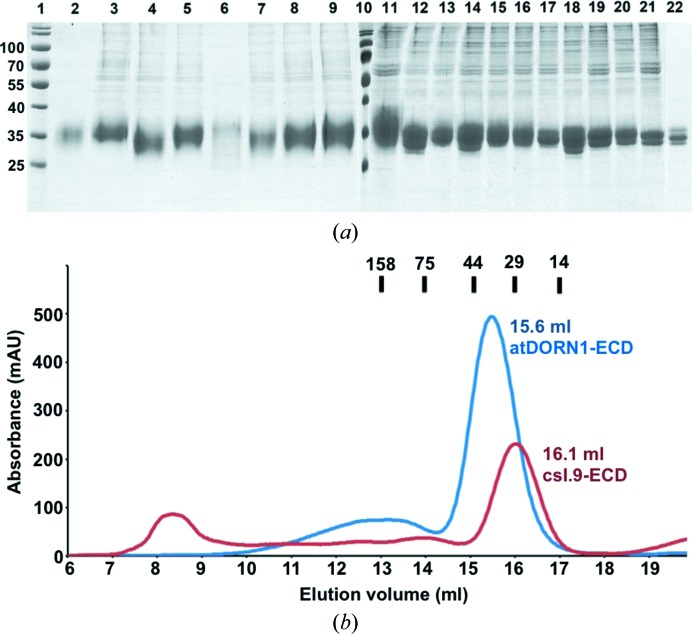
(*a*) Gel electrophoresis of purified atDORN1-ECD, atDORN1-ECD mutant and csI.9-ECD proteins resolved and analyzed by SDS–PAGE. Protein molecular-weight marker is in lanes 1 and 10 (labeled in kDa), while lane 2 contains atDORN1-ECD, lane 3 atDORN1-ECD-N56D, lane 4 atDORN1-ECD-N124D, lane 5 atDORN1-ECD-N128D, lane 6 atDORN1-ECD-N181D, lane 7 atDORN1-ECD-N204D, lane 8 atDORN1-ECD-N225D, lane 9 atDORN1-ECD-N232D, lane 11 atDORN1-ECD, lane 12 atDORN1-ECD-N124N128D, lane 13 atDORN1-ECD-N124N204D, lane 14 atDORN1-ECD-N124N225D, lane 15 atDORN1-ECD-N128N204D, lane 16 atDORN1-ECD-N204N225D, lane 17 atDORN1-ECD-N124N128N204D, lane 18 atDORN1-ECD-N124N128N225D, lane 19 atDORN1-ECD-N124N204N225D, lane 20 atDORN1-ECD-N128N204N225D, lane 21 atDORN1-ECD-N124N128N204N225D and lane 22 csI.9-ECD. SDS–PAGEs were performed on 12%(*w*/*v*) gel and were stained with Coomassie Brilliant Blue. (*b*) Chromatogram showing the elution profiles of atDORN1-ECD (blue) and csI.9-ECD (orange) from size-exclusion chromatography on a Superdex 200 10/30 column. The major peaks at the retention volumes of 15.6 and 16.1 ml correspond to atDORN1-ECD and csI.9-ECD monomers, respectively. Molecular-weight standards are indicated in kDa at the top of the profiles.

It is challenging to express and crystallize extracellular secreted proteins owing to glycosylation. We expressed the extracellular domain (ECD) of *A. thaliana* DORN1 (residues 25–277; atDORN1-ECD) in a baculovirus insect-cell secretion expression system with an average yield of 10 mg of protein per litre of cells. The atDORN1 gene encoding residues 25–277 was fused to the secretion signal sequence of baculovirus gp67 glycoprotein and then cloned into a modified pFastBac1 vector (Table 1 ▸). The secreted protein was first purified by nickel-affinity chromatography using an engineered six-histidine tag at the carboxyl-terminus of the protein, and was further purified by size-exclusion chromatography in a buffer consisting of 20 m*M* Tris–HCl pH 8.0, 100 m*M* NaCl. The recombinant atDORN1-ECD was concentrated to about 10 mg ml^−1^, mixed with 5 m*M* freshly prepared ATP and incubated at 277 K for 1 h. The atDORN1-ECD/ATP mixture was subjected to extensive crystallization screening. The recombinant DORN1 protein has a molecular weight that is about 6 kDa larger than that predicted from its amino-acid sequence (Fig. 1 ▸ *a*, lanes 2 and 11), and this is presumably caused by glycosylation. We speculated that glycosylation of the protein may introduce flexibility and heterogeneity into the protein, which render it refractory to crystallization. In order to overcome this difficulty, the purified protein was digested with either PNGase F or Endo H (New England Biolabs), which remove or trim N-glycosylation chains on the protein. Briefly, 20 µg protein was incubated with 1 µl PNGase F (500 units) in a buffer consisting of 20 m*M* Tris–HCl pH 7.5 at 277 K overnight. For Endo H reaction, 20 µg protein was mixed with 1 µl Endo H (500 units) in buffer consisting of 50 m*M* sodium acetate at 277 K overnight. The digested protein was then purified by size-exclusion chromatography for further crystallization trials.

#### 2.1.2. Deleting putative glycosylation sites on atDORN1-ECD by site-directed mutagenesis   

Seven asparagine residues (Asn56, Asn124, Asn128, Asn181, Asn204, Asn225 and Asn232) have been predicted to be putative N-glycosylation sites on atDORN1 (*GlycoEP* online server; Chauhan *et al.*, 2013 ▸). The O-glycosylation sites of plant proteins are difficult to predict owing to a lack of understanding of the biological process (Wilson, 2002 ▸); we therefore focused our mutational studies only on the N-glycosylation sites in order to reduce protein glycosylation. Seven single-site mutants and ten multisite mutants (N124N128D, N124N204D, N124N225D, N128N204D, N204N225D, N124N128N204D, N124N128N225D, N124N204N225D, N128N204N225D and N124N128N204N225D) were constructed by site-directed mutagenesis with PfuUltra High-Fidelity DNA Polymerase AD (Agilent). The mutants were expressed and purified for crystallization trials using the procedure described above.

### 2.2. Expression, purification and crystallization of csI.9-ECD protein   

*Camelina sativa* lectin receptor kinase I.9 (csLecRK-I.9, csI.9-ECD) shares 90% overall sequence identity with the *Arabidopsis* DORN1 protein and was identified in a *BLAST* search (Supplementary Fig. S1). The extracellular domain (residues 34–293) of csI.9 (csI.9-ECD) was purified as described above. The average yield of the recombinant protein was about 10 mg per litre of Hi5 insect cells. The recombinant csI.9-ECD protein was concentrated to 5 mg ml^−1^, 5 m*M* freshly prepared ATP was added and the mixture was incubated at 277 K for 1 h before crystallization. For each protein, we manually set up initial screens with 960 different conditions in 96-well sitting-drop plates (Intelli-Plate 96-3 LVR, Hampton Research). The crystallization screen solutions consisted of 11 separate kits: Crystal Screen (conditions 1–48) and Crystal Screen 2 (48 conditions) from Hampton Research and The PEGs Suite, The PEGs II Suite, The MPD Suite, The AmSO_4_ Suite, The Cations Suite, The Anions Suite, The ComPAS Suite, The pHClear Suite and The ProComplex Suite from Qiagen. Each of the Qiagen kits contained 96 conditions. The crystallization plates were set up at room temperature (298 K) and were kept at 291 K. csI.9 crystals were observed in condition C12 of The PEGs Suite (0.1 *M* MES pH 6.5, 15% PEG 20 000). The best crystals grew to around 0.2 mm in the largest dimension in 7 d (Table 2 ▸).

### 2.3. Data collection and processing   

For data collection, all crystals were flash-cooled in crystallization reservoir solution supplemented with 30%(*v*/*v*) glycerol. Diffraction data were collected on the 22-ID (SER-CAT) beamline at the Advanced Photon Source (APS) with a Rayonix (MAR) 300HS high-speed CCD detector using the remote data-collection software *SERGUI* running through *NX Client*. All diffraction data were processed with *HKL*-2000 and scaled with *SCALEPACK*. The statistics are shown in Table 3 ▸.

## 3. Results and discussion   

The measured molecular weight of the recombinant atDORN1-ECD protein was 35 kDa, which is 6 kDa larger than its predicted molecular weight (Fig. 1 ▸ *a*, lanes 2 and 11). Size-exclusion chromatography indicated that it exists as a monomer in solution (Fig. 1 ▸ *b*). Secreted proteins have been known to possess glycosylation that adds additional molecular weight to the expressed recombinant proteins. We speculated that the higher apparent molecular weight of atDORN1-ECD can be attributed to glycosylation modifications. Glycosylation increases the stability of many secreted proteins by protecting them against degradation by proteases and may also be required for proper protein folding (Gahmberg & Tolvanen, 1996 ▸; Wormald & Dwek, 1999 ▸; Rudd *et al.*, 1995 ▸; Imperiali & O’Connor, 1999 ▸; Mitra *et al.*, 2006 ▸; Braakman & Bulleid, 2011 ▸). In some cases glycosylation is required for ligand binding (Olson & Lane, 1989 ▸; Chamorey *et al.*, 2002 ▸; Opdenakker *et al.*, 1995 ▸; Standley & Baudry, 2000 ▸). However, we realise that glycosylation may present a problem in protein crystallization. Long oligosaccharide chains conjugated to the secreted proteins may have intrinsic flexibility, or owing to overexpression secreted proteins may have heterogeneous glycosylation. Both cases are detrimental to protein crystallization. Consequently, deletion of the glycosylation sites or trimming the long sugar chains may help to reduce the flexibility of the protein and facilitate crystallization. Indeed, we observed a smeared band pattern for the recombinant atDORN1-ECD protein, which indicates a heterogeneous nature of the protein (Fig. 1 ▸ *a*, lanes 2 and 11). We subjected the recombinant atDORN1-ECD protein to extensive crystallization trials; no reproducible protein crystals were obtained.

In order to circumvent this difficulty, we attempted to delete the N-glycan chains by digesting the protein with PNGase F (Tarentino *et al.*, 1989 ▸) or to trim the sugar chains with Endo H (Chien *et al.*, 1977 ▸). PNGase F is an amidase that cleaves between the innermost GlcNAc and asparagine residues, removing all N-linked carbohydrates, whereas Endo H cleaves between the GlcNAc molecules in the di-*N*-acetyl­chitobiose core of oligomannose and hybrid-type N-glycans, leaving a single GlcNAc molecule at each glycosylation site. However, the treated proteins tended to aggregate and were not suitable for protein crystallization. We next systematically mutated each of the seven predicted N-glycosylation sites (Asn56, Asn124, Asn128, Asn181, Asn204, Asn225 and Asn232) to aspartate. These mutants were all successfully expressed and purified with a similar yield to the wild-type protein, with the exception of the N181D mutant (Fig. 1 ▸ *a*, lane 6), indicating that Asn181 might be essential for the protein folding or stability of DORN1. Both the N124D and N204D mutants had a significant downshift on SDS–PAGE (Fig. 1 ▸ *a*, lanes 4 and 7), indicating that Asn124 and Asn204 are modified by glycosylation in the wild-type protein. The N128D and N225D mutants had a less significant downshift (Fig. 1 ▸ *a*, lanes 5 and 8), showing that these two resides have shorter modified sugar chains in the wild-type DORN1 protein. The other mutants had a negligible downshift, indicating that they are not glycosylated in the recombinant atDORN1-ECD protein. We then focused on Asn124, Asn204, Asn128 and Asn225 to generate double-site or multisite mutants to further reduce glycosylation of atDORN1-ECD (N124N128D, N124N204D, N124N225D, N128N204D, N204N225D, N124N128N204D, N124N128N225D, N124N204N225D, N128N204N225D and N124N128N204N225D; Fig. 1 ▸ *a*, lanes 12–21). The expression yields of the mutants vary from 20 to 80% of that of the wild-type atDORN1-ECD protein. Most of them showed a sharper band pattern on SDS–PAGE, which indicates less glycosylation of the proteins. Unfortunately, none of the modified proteins yielded reproducible protein crystals for X-ray crystallographic analysis.

**Figure 2:**
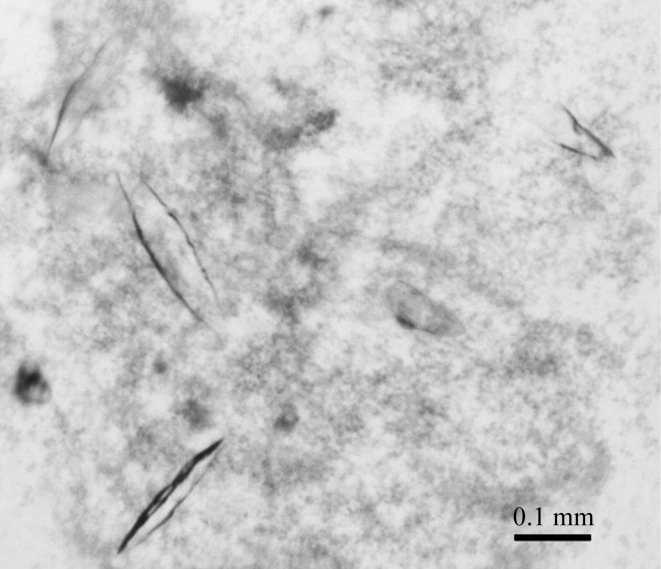
Crystals of csI.9-ECD grown in 100 m*M* MES pH 6.5, 15% PEG 20 000.

**Figure 3:**
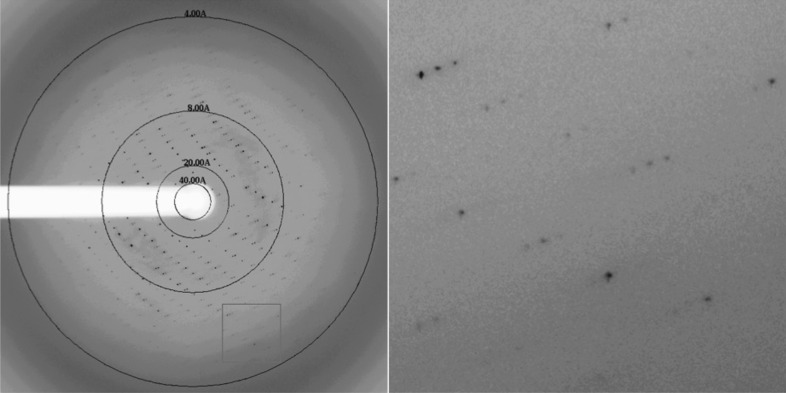
X-ray diffraction data from a csI.9-ECD crystal. The boxed high-resolution area is magnified on the right.

We conducted a *BLAST* search to find proteins homologous to atDORN1 for further crystallographic studies. *C. sativa* lectin receptor kinase I.9 (csLecRK-I.9 or csI.9) was identified to share 90% overall protein sequence identity and 85% identity with atDORN1 and atDORN1-ECD, respectively (Supplementary Fig. S1). We expressed the ECD of csI.9 (Fig. 1 ▸ *a*, lane 22, and Fig. 1 ▸ *b*), which has an apparent molecular weight of 33 kDa on SDS–PAGE; this is 6 kDa larger than the calculated molecular weight (27 kDa) and is likely owing to glycosylation. The purified protein was crystallized successfully, with a size of about 0.2 mm in the largest dimension (Fig. 2 ▸). These crystals diffracted to 4.6 Å resolution (Fig. 3 ▸), and a diffraction data set was collected on the 22-ID (SER-CAT) beamline of the Advanced Photon Source (APS). The crystal belonged to space group *C*222 or *C*222_1_ (*C* ortho­rhombic unit cell), with unit-cell parameters *a* = 94.7, *b* = 191.5, *c* = 302.8 Å (Table 3 ▸). Eight csI.9-ECD molecules are estimated to be present in each asymmetric unit based on a Matthews coefficient calculation (http://csb.wfu.edu/tools/vmcalc/vm.html). In addition to glycosylation, the nature of the large unit-cell dimensions as a result of the presence of multiple copies of the csI.9-ECD protein molecule in the asymmetric unit may further weaken the diffraction limit of the crystal.

In order to verify whether the crystals were indeed the target recombinant protein, we isolated the crystals and resolved them by SDS–PAGE (Supplementary Fig. S2). In contrast to the three bands for the control protein, there are two bands for the crystal, which correspond to the middle major band and the top minor band of the sample. In addition, there is a top band that is not present in recombinant csI.9-ECD. We speculated that these three bands of the expressed protein are differential glycosylation products of csI.9-ECD, while the top band of the crystal is disulfide-bond cross-linked csI.9-ECD protein arising from oxidation during crystallization that was not completely reduced during sample preparation for SDS–PAGE. To test these speculations, we excised these three bands from the crystal sample, digested then with trypsin and analyzed the tryptic peptides by mass spectrometry. The results clearly showed that all three bands are products of the same csI.9-ECD protein.

We conducted a *BLAST* search with the amino-acid sequence of csI.9-ECD against the PDB; the structure with the highest homology to the crystallized csLecRK-I.9-ECD domain is a vegetative lectin from the legume *Dolichos biflorus* (PDB entry 1g8w, 74/249 residues, 30% identity). Interestingly, four molecules of this protein form a tetramer in the asymmetric unit (Buts *et al.*, 2001 ▸). We speculated that there are two csI.9 tetramers in the asymmetric unit of the crystal, which is consistent with Matthews coefficient calculation. In order to validate this, we conducted self-rotation function (SRF) and native Patterson analyses. The native Patterson analysis did not yield any significant peaks. However, the SRF resulted in two prominent peaks at θ = 45° and ψ = 90° along the *y* axis of the χ = 180° (twofold non­crystallographic symmetry; NCS) section (Supplementary Fig. S3), which could indicate a noncrystallographic relation between two tetramers. The two observed peaks at θ = 90° and φ = 0° along the *x* axis on the χ = 90° (fourfold NCS) section is likely to be owing to the interactions between twofold NCS and twofold crystallographic symmetry (Borhani *et al.*, 1999 ▸). The absence of significant peaks in the χ = 120° (threefold NCS) and χ = 60° (sixfold NCS) sections further support the presence of eight molecules in the asymmetric unit.

We are currently attempting molecular-replacement approaches using this homologous structure as a search model to determine the structure of csI.9-ECD. In the meantime, we are also optimizing the crystallization conditions to improve the diffraction resolution limit to better than 3 Å. In addition, we will employ the deglycosylation approaches used for expressing atDORN1-ECD to optimize the csI.9-ECD protein for better crystallization. Indeed, five of the seven putative N-glycosylation sites are conserved in csI.9 (Supplementary Fig. S1). The presence of two protein bands in the crystals indicates that heterogeneous glycosylation might have contributed to the low diffraction quality of the obtained crystals. These optimization strategies will lead to the eventual structural determination of csI.9-ECD.

## Supplementary Material

Supplementary figures.. DOI: 10.1107/S2053230X16014278/dp5095sup1.pdf

## Figures and Tables

**Table 1:** Macromolecule-production information

Source organism	*A. thaliana*	*C. sativa*
DNA source	The *Arabidopsis* Information Resource (TAIR)	DNA synthesis
Forward primer	5′-GCGGATCCCACAAGCTTTGTCTATGAAAGCTTCCT-3′†	5′-CATGGCGGCCGCAGTCAACAAGAGACAAGGTTTGTCTA-3′‡
Reverse primer	5′-CATGGCGGCCGCCTAGTGGTGATGGTGGTGGTGAGGAACTTCAGGAAGTTTTGAGATATC-3′‡	5′-GCGGATCCCTAGTGGTGATGGTGGTGGTGTGGATGAGGAACTTTAGGAAGTTTTG-3′†
Cloning vector	pFastBac1	pFastBac1
Expression vector	Baculovirus	Baculovirus
Expression host	Insect cells (High Five)	Insect cells (High Five)
Complete amino-acid sequence of the construct produced§	ADPTSFVYESFLDRQNLYLDKSAIVLPSGLLQLTNASEHQMGHAFHKKPIEFSSSGPLSFSTHFVCALVPKPGFEGGHGIVFVLSPSMDFTHAESTRYLGIFNASTNGSSSYHVLAVELDTIWNPDFKDIDHNHVGIDVNSPISVAIASASYYSDMKGSNESINLLSGNPIQVWVDYEGTLLNVSVAPLEVQKPTRPLLSHPINLTELFPNRSSLFAGFSAATGTAISDQYILWWSFSIDRGSLQRLDISKLPEVPHHHHHH	ADGGRSQQETRFVYESFLDQENLYIDKSATVLPSGILRLTNASEHQMGHAFHKKPLEFSSSGPLSFSTHFVCALVPKPRVEGGHGIAFVLSPSMDFTHAESTRYLGIFNASTSGSSSYHVLAVELDTIWNPDFKDIDHNHVGIDVNSPISVAIASASYFSDIKGSHERVDLLSGRPIQVWVDYEGTMLNVSIAPLKVQKPSRPLLSHPINLSKFFPNRSRLFVGFSASTGTAISDQYILWWSFSTRRGSLQGFDISKLPKVPHPHHHHHH

† Contains a BamHI site.

‡ Contains a NotI site.

§ The sequences derived from the cloning vector and the engineered C-terminal six-histidine tags are underlined.

**Table 2:** Crystallization of csI.9-ECD protein

Method	Vapour diffusion
Plate type	Hanging drop
Temperature (K)	291
Protein concentration (mg ml^−1^)	5
Buffer composition of protein solution	20 m*M* Tris pH 8.0, 100 m*M* NaCl
Composition of reservoir solution	100 m*M* MES pH 6.5, 15% PEG 20 000
Volume and ratio of drop	4 µl drop, 1:1 protein:well solution
Volume of reservoir (µl)	400

**Table 3:** X-ray crystallographic statistics of a csI.9-ECD crystal Values in parentheses are for the outer shell.

Diffraction source	Advanced Photon Source
Wavelength (Å)	1.0000
Rotation range per image (°)	0.5
Exposure time per image (s)	2
Space group	*C*222 or *C*222_1_
Unit-cell parameters (Å, °)	*a* = 94.7, *b* = 191.5, *c* = 302.8, α = β = γ = 90.0
Resolution range (Å)	50–4.60 (4.68–4.60)
Total reflections	11377 (566)
No. of unique reflections	5418 (283)
Completeness (%)	71.6 (71.4)†
Multiplicity	2.1 (2.0)
〈*I*/σ(*I*)〉	17.1 (2.1)
*R*_r.i.m._	0.081 (0.386)
Mosaicity (°)	0.95

† Owing to rapid decay of the crystal during X-ray diffraction, the data completeness was not able to reach more than 80% with the current crystal. The crystal diffraction is not anisotropic, and the decay of the collected data is mostly owing to X-ray damage to the crystal. For the only crystal from which we were able to collect a data set, 80 frames of data which cover an 80° angle were successfully collected and processed. After 80° the rest of the data had significant decay, the inclusion of which will further lower the overall resolution and quality of the data without providing a significant increase in completeness. The completeness of the processed data is consistently about 72% in all resolution shells.
