# Supplementary material for: X-ray crystallographic studies of the extracellular domain of the first plant ATP receptor, DORN1, and the orthologous protein from Camelina sativa
Source: Acta Crystallogr F Struct Biol Commun. 2016 Sep 22;72(Pt 10):782–7. doi: 10.1107/S2053230X16014278 (PMC5053164; doi:10.1107/S2053230X16014278)
Supplement: Supplementary file 1 [file f-72-00782-sup1.pdf]

STRUCTURAL BIOLOGY
COMMUNICATIONS

Volume 72 (2016)

Supporting information for article:

X-ray crystallographic studies of the extracellular domain of the first plant ATP receptor, DORN1, and its orthologous protein in *Camelina sativa*

Zhijie Li, Sayan Chakraborty and Guozhou Xu

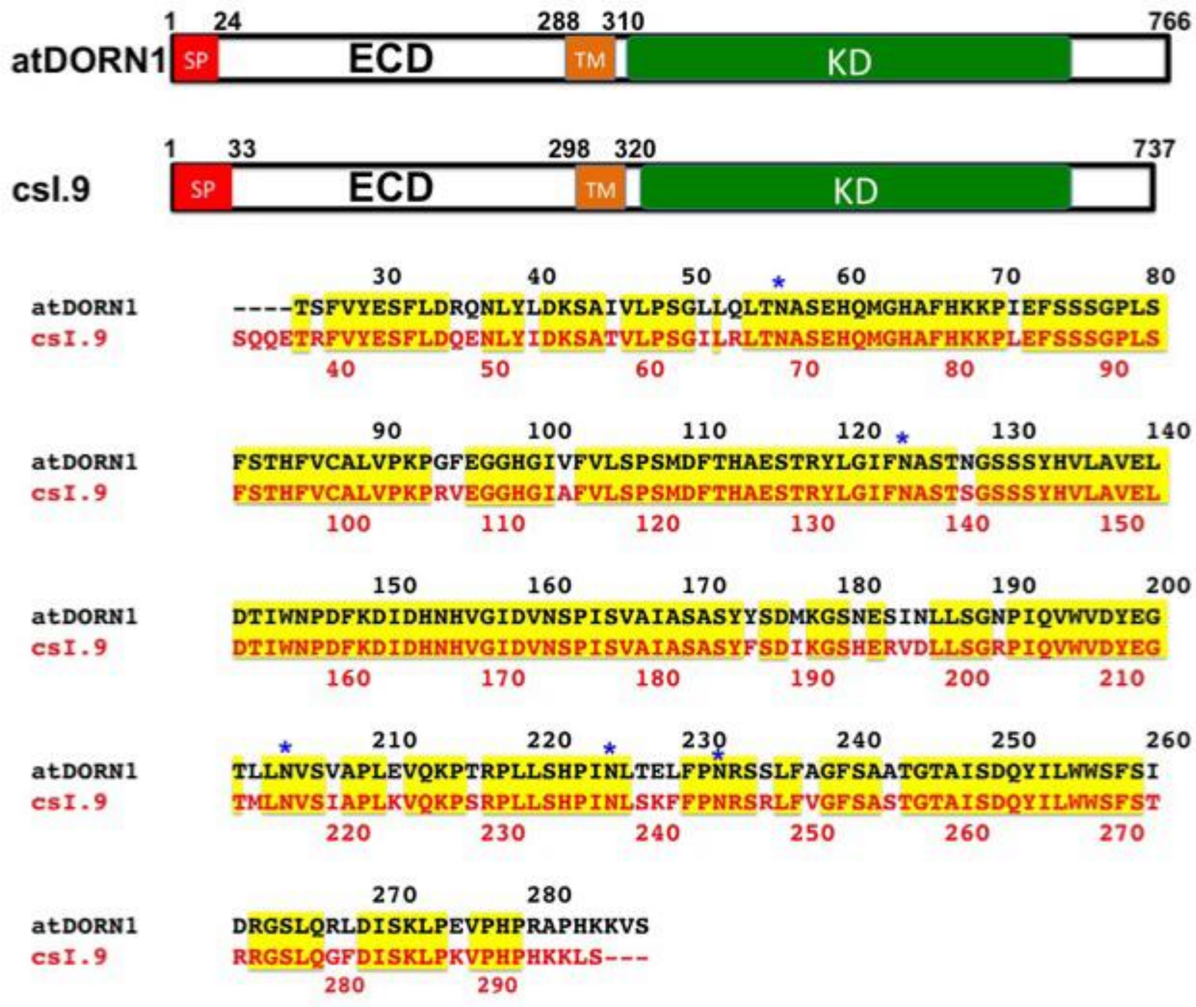

Figure S1 Domain organizations of the atDORN1 and csI.9 proteins. SP: Signal Peptide. ECD: ExtraCelluar Domain. TM: TransMembrane domain. KD: Kinase Domain. The domain boundaries are labeled on the top of each diagram. The sequence alignment between the ECD of atDORN1 (aa 25-287) and csI.9 (aa 34-297) are shown below the diagrams. The overall sequence identity between these two proteins is 90%, while the ECDs of both proteins share 85% sequence identity. The identical residues are contained in yellow boxes, with residue numbers of atDORN1 shown on the top of the sequence, and that of csI-9 below its sequence. The first digit of each number aligns with the indicated residue. The sequence of atDORN1 is colored in black, and that of csI.9 in red.

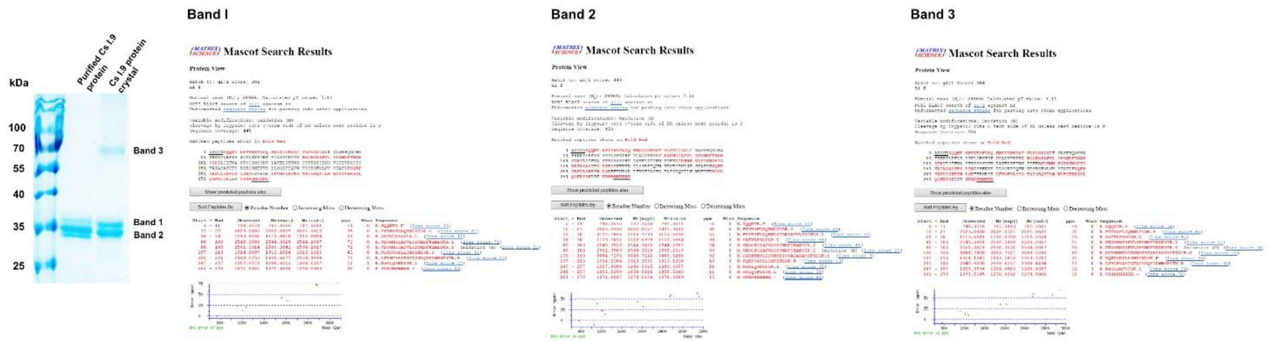

Figure S2 Mass spectrometry analysis of the csI.9-ECD crystals. The sequences derived from the cloning vector and the engineered C-terminal 6-histidine tags are underlined.

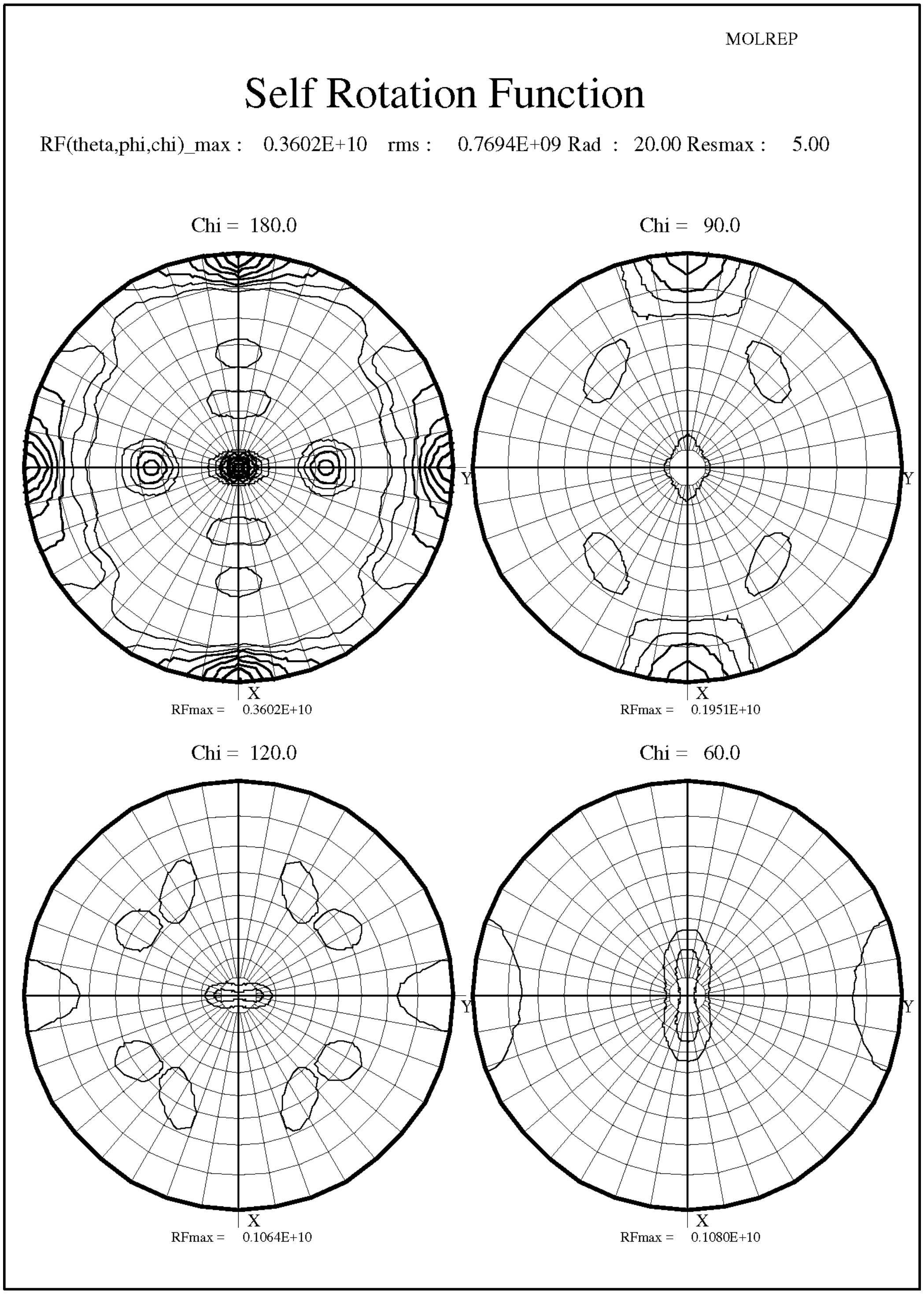

Figure S3 Self Rotation Function of the csI.9-ECD X-ray diffraction data calculated by MOLREP with the chi angles shown. Since the unit cell is C orthorhombic, the X, Y, Z axes correspond to the crystallographic a, b, c axes respectively. The Z axis is perpendicular to the sections along the center.
